# Advocacy Through Partnerships: Data, Demographics, and Decisions

**DOI:** 10.1093/geroni/igaa057.1862

**Published:** 2020-12-16

**Authors:** Briana Sisofo, Anne Asman

**Affiliations:** 1 University of Utah, Park City, Utah, United States; 2 University of Utah, Salt Lake City, Utah, United States

## Abstract

The Summit County Aging Alliance (SCAA) in Park City, Utah is representative of state government, county and city government(s), private citizens, land developers, Senior Center attendees, national and local associations, non-profit support organizations, home health agencies, colleges and universities, recreation centers, hospital administrators and area associations on aging. The focus of the Alliance has been to provide a forum for critical listening and discussion. Data from a survey to determine the ‘real’ needs and vision of the older adult community provided perspective from more than 100 older adults representing diverse ethnic and socio-economic backgrounds. This work is now providing a benchmark from which both the city and county governments in Summit County are creating their strategic plans, and the Alliance has become the official voice of the community’s older adults.

